# Is the information available on the Web influencing the way parents see ENT surgical procedures?

**DOI:** 10.1016/S1808-8694(15)30490-0

**Published:** 2015-10-19

**Authors:** João Flávio Nogueira, Diego Rodrigo Hermann, Maria Laura Solferini Silva, Fábio Pires Santos, Shirley Shizue Nagata Pignatari, Aldo Cassol Stamm

**Affiliations:** 1 ENT Resident; 2 ENT; 3 ENT Resident; 4 ENT Resident; 5 Head of the ENT Department and ENT and Speech and Hearing Therapy Center of São Paulo - Hospital Professor Edmundo Vasconcelos; 6 Head of the Speech and Hearing Therapy Center of São Paulo - Hospital Professor Edmundo Vasconcelos. São Paulo ENT Center, Hospital Professor Edmundo Vasconcelos

**Keywords:** surgery, information, internet

## Abstract

**Introduction and Aims:** the Internet is the world's fastest growing source of health related information. Parents and guardians are increasingly turning to the Internet for information about their children's medical conditions and treatments. This paper has the following objectives 1) determine the prevalence of web searches for medical information performed by parents/guardians of children undergoing ENT surgery in a private hospital of Sao Paulo, Brazil. 2) assess whether the gathered information influenced the parents/guardians' decision on the treatment/surgery.

**Method:**

questionnaire voluntarily responded by 132 parents/guardians of children submitted to ENT surgery in a private hospital in Sao Paulo, Brazil.

**Conclusions:**

117 parents/guardians (90%) used the Internet to search for information on the disease and surgical treatment of the children. Only 12 parents/guardians (10%) discussed the information with the physician assigned to perform the surgical procedure. 91 parents/guardians (78%) said that the information had impact upon the decision to have surgery performed on their children.

## INTRODUCTION

The Internet is the fastest-growing source of health-related information. A study conducted by Pew Internet & American Life Project observed that 93 million American adults have looked for information on health and specific disease treatment. That same poll found that 73% of the people looking for information on the Web believed they were better informed after reading the material they found in the Internet^1^.

There is no data in Brazil about the prevalence of Internet searches for health related information. The only available data is on access to the Web. According to a survey conducted by IBOPE (Instituto Brasileiro de Opinião Pública e Estatística - Brazilian Institute of Public Opinion and Statistics) in March of 2008 21.1 million Brazilians had access to the Internet^2^.

That same survey indicated that Web access in Brazil grows at a monthly rate of 1 million new users, thus stressing the relevance of the Internet as a major source of information in general and on health care more specifically^2^.

Parents and guardians are increasingly looking at the Web as a source of information on their children's conditions and therapies^3^. Recent studies have however questioned the quality and accuracy of such information^4^, ^5^, ^6^. Studies done in Brazil are yet to show the degree of influence the information collected on the Web has had on parents and guardians as they look up physicians, health care services, and disease treatment options.

Unfortunately, most physicians are ill prepared to discuss with their patients the information they gather on the Web. Many will simply and utterly regard such information as rubbish, warning their patients to refrain from using the Web with this purpose, without however being able to refer them to web sites with reliable content^7^. This negative approach may lead to patient suspicion.

In spite of its limitations, the Internet came to stay as an attractive source of information. Knowledge on Web use may aid physicians in advising their patients on how to best use this tool, apart from improving the physician/patient relationship, introducing more and better discussions on the obtained medical information.

## OBJECTIVES

This paper has two objectives:

1) Determine the prevalence of Web searching for medical information among parents/guardians of children undergoing ENT surgery at a private hospital in São Paulo, Brazil.
2) Assess whether the gathered information impacted the decisions of parents/guardians to allow their children to undergo surgery.

## MATERIALS AND METHOD

A questionnaire (Annex 2) was developed based on other questionnaires found in the literature8-10. The questionnaire presents 20 multiple choice questions, some of which followed by soma space for comments by respondents.

Questionnaires were handed out to parents/guardians of children of both genders aged between 2 and 14 years advised to have ENT surgery at a private hospital in São Paulo, Brazil, in the months of January to October of 2007.

This study was approved by the hospital's ethics committee under permit 012/07. Results were submitted to statistical analysis.

## RESULTS

All parents/guardians asked to answer the questionnaire did so (n=132). Respondent age ranged between 18 and 66 years of age (mean age of 42 years).

Eighty-three percent of the respondents were mothers to the patients (n=110). Twelve (9%) respondents had primary level education while 96 (73%) had higher education diplomas. Internet access was reported by 130 (98%) respondents (Table 1).

**Table 1:** Demographic data (n=132)

**Surgery performed on patients**	
Adenotonsillectomy	61 (46%)
Other procedures*	71 (54%)
Age of parents/guardians (mean)	18-66 (42)
**Relation to Patient**	
Mother	110 (83%)
Father	18 (14%)
Other	4 (3%)
**Level of Education**	
Completed higher education degree	96 (73%)
Complete secondary education degree	34 (26%)
Complete primary education degree	2 (1%)
**Internet Access**	
Yes	130 (98%)
No	2 (2%)

There was no significant statistical difference between parents and guardians with higher education degrees and complete and incomplete secondary education degree in terms of Internet access (p<0.03).

* Other procedures include adenoidectomy and/or tonsillectomy and/or tympanotomy and/or turbinate cauterization etc.

There was no statistically significant difference between parents/guardians with complete or incomplete higher education degrees in terms of web access (p<0.03).

One hundred and seventeen (90%) of the 130 respondents with Internet access used the Web to search for information on their children's condition. Out of this group, 27 (23%) reported the information was easy to understand and find, while 88 (75%) found that only some of the information was easy to understand. Only 2 (2%) could not understand the information found on the Web (Table 2).

**Table 2:** Questionnaire - searching for disease information on the Web

**Did you look for information on the Web on the condition of your child/guardian? (n=130)**	
Yes	117 (90%)
No	13 (10%)
**Was the information readily accessible and easy to understand? (n=117)**	
Yes	27 (23%)
Algumas Yes	88 (75%)
No	2 (2%)
**Was the information useful? (n=117)**	
Yes	25 (21%)
Algumas Yes	63 (54%)
No	29 (25%)
**Did the information collected on the Web impact your decision regarding the proposed treatment? (n=117)**	
Yes	91 (78%)
No	26 (22%)
**Did you discuss such information with your physician? (n=117)**	
Yes	12 (10%)
No	105 (90%)

Those who used the Web to search for data on their children's condition have also looked up the proposed surgical treatment. Similarly to what had been found before, 23% reported the information was easy to understand and find, 75% found that only part of the information was easy to understand, and 2% were not able to understand the information they found (Table 3).

**Table 3:** Questionnaire - w searching for surgery information on the Web

**Was the information readily accessible and easy to understand? (n=117)**	
Yes	27 (23%)
Some	88 (75%)
No	2 (2%)
**Was the information useful? (n=117)**	
Yes	25 (21%)
Some	63 (54%)
No	29 (25%)
**Did the information collected on the Web impact your decision regarding the proposed treatment? (n=117)**	
Yes	91 (78%)
No	26 (22%)
**Did you discuss such information with your physician? (n=117)**	
Yes	12 (10%)
No	105 (90%)

Only 12 (10%) parents/guardians discussed the information acquired over the Web with the physician assigned to perform surgery on their children. However, 91 (78%) stated that the information they found on the Internet impacted their decisions around the procedures to be performed on their children.

**Graph 1:**
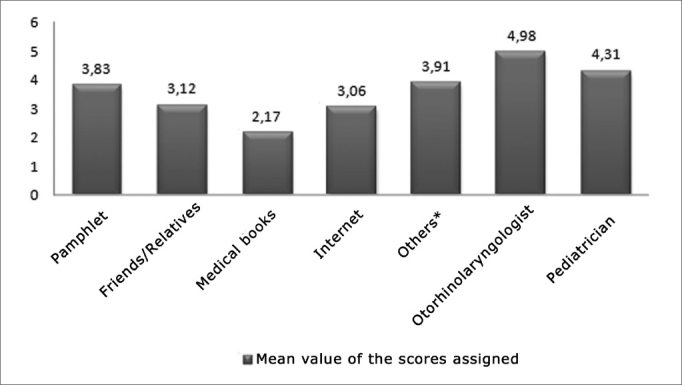
Mean scores assigned by parents/guardians to 7 different means of medical information according to relevance and information on diagnosis and surgical treatment. Scale going from 1 to 5 (1 = least important; 5 = most important). * Other health care workers such as nurses, dentists, and pharmacists.

In the questionnaire, respondents (n=132) were asked to put in order of relevance seven means of medical communication in the realm of diagnosis and surgical treatment (Graph 1).

Although most respondents were impacted by the information they found on the Web, the Internet was ranked as one of the least important of all sources of medical information.

**Questionnaire:**

1) Date: _____ / _____ / _____
2) Surgery patient was submitted to: ________________________
3) Relation to patient:
  ( ) Father
  ( ) Mother
  ( ) Grandparent
  ( ) Other
4) Education:
  ( ) Complete primary education degree
  ( ) Complete secondary education degree
  ( ) Higher education degree
  ( ) Other
5) Do you have access to the Internet?
  ( ) Yes
  ( ) No
6) Did you search the Internet for information on your child's condition?
  ( ) Yes
  ( ) No
7) Was the information readily accessible and easy to understand?
  ( ) Yes
  ( ) Some
  ( ) No
8) Did you find the information useful?
  ( ) Yes
  ( ) Some
  ( ) No
9) Did the information you collect on the Internet impact the decisions you made upon the treatment given to your child?
  ( ) Yes
  ( ) No
10) Did you discuss the information collected on the Internet with your child's physician?
  ( ) Yes
  ( ) No
11) Did you look for information on the treatment prescribed to your child's condition on the Internet?
  ( ) Yes
  ( ) No
12) Was the information readily accessible and easy to understand?
  ( ) Yes
  ( ) Some
  ( ) No
13) Did you find the information useful?
  ( ) Yes
  ( ) Some
  ( ) No
14) Did the information you collect on the Internet impact the decisions you made upon the treatment given to your child?
  ( ) Yes
  ( ) No
15) Did you discuss the information collected on the Internet with your child's physician?
  ( ) Yes
  ( ) No
16) Check the search engines you used to find medical information on the Web:
  ( ) Google
  ( ) Yahoo
  ( ) Website of the Brazilian Association of Otorhinolaryngology and Head and Neck Surgery
  ( ) Website recommended by your child's physician
  ( ) Others; please list them: ________________________________
17) Assign a score to the following sources of information in terms of their importance: (1 = least important; 5 = most important):
  ( ) Pamphlets
  ( ) Friends/Relatives
  ( ) Medical literature
  ( ) Internet
  ( ) Other health care workers
  ( ) ENT doctor
  ( ) Pediatrician
18) Will you use the Internet in the future as a source for medical information?
  ( ) Yes
  ( ) No
19) Would you like to have reliable sources of medical information recommended to you by your child's physician?
  ( ) Yes
  ( ) No
20) Would you like to discuss your doubts or get in touch with your child's physician through e-mail?
  ( ) Yes
  ( ) No

**Graph 2:**
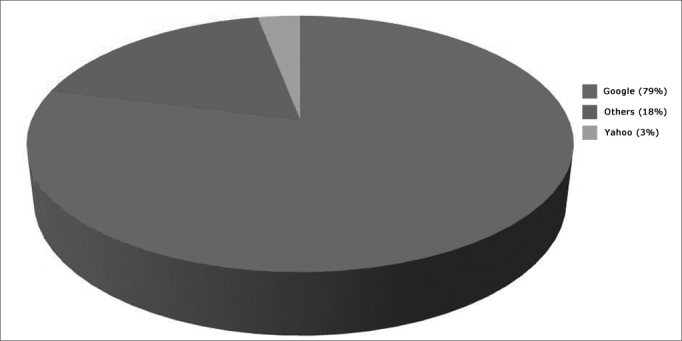
Websites most frequently accessed by parents/guardians in search for medical information:

In the questionnaire, respondents were asked to list Web pages and search engines they used to gather for medical information. The top three sources were: Google 79% (n=92); others 18% (n=21); Yahoo 3% (n=4) (Graph 2). None of the respondents used the website of the Brazilian Association of Otorhinolaryngology and Head and Neck Surgery (Associação Brasileira de Otorrinolaringologia e Cirurgia Cérvico Facial - ABORLCCF) or websites recommended by their ENT doctors.

Not all respondents searched the Web for information on their children's condition and treatment, but 97% of them said they would resort to the world wide web if necessary.

All respondents (100%) would like have access to reliable information from websites recommended by their children's physician. Additionally, 74% of the parents/guardians mentioned a desire to have their doubts and questions discussed with their children's ENT doctor through e-mail.

## DISCUSSION

Internet searching for medical information has grown quite substantially within the past few years. In 2003, 85% of the physicians surveyed in the United States saw patients who asked questions derived from their Web searches^7^. In spite of the lack of information on this matter in Brazil, there is a sense that ENT doctors are being more questioned by patients as a result of their searches on the Internet.

In our study the vast majority of respondents (98%) had access to the Internet, while 90% of them looked up their children's condition and treatment. The fact that this study was carried out in a private hospital should by itself be carefully considered, as more affluent and better educated people have higher Internet access penetration rates^2^. Nonetheless, a great deal of the ENT doctors in Brazil sees patients in their private practice, as observed in the poll published by ABORL-CCF^8^.

As a sequel to this study, the same questionnaire will be distributed to patients and guardians of children undergoing ENT surgery in public hospitals to verify whether significant differences will be found in comparison to private hospitals.

Despite the growing number of searches for medical information, traditional sources still prevail among those searching for information on diseases and treatment. A survey conducted in the United States shows that physicians still are the primary source of information for parents/guardians on the conditions and treatment modes for their families^9^. The same was found in our survey. Parents/guardians stated that their children's physician was the most important source of preoperative information among the given options.

Surprisingly enough, medical literature was referred to as the least reliable source, possibly due to their intricate language and limited access.

Although they were seen as the most reliable source of information, physicians must be aware of the growing use and trust patients are assigning to the Internet. The Web will keep on growing in terms of popularity and relevance. In our survey, 97% of the respondents - including those who claimed not to have searched the Web for medical information - reported they would do it in the future.

However, the most important finding of our study was the degree with which the information gathered on the Web impacted the decisions made by parents/guardians in regards to the procedure their children were to undergo. Ninety-one parents/guardians (78%) reported that the information they found impacted their decision on the procedure to be performed on their children. That is both impressive and troubling, as parents/guardians may have had access to unreliable information on the Internet.

American studies have shown that the medical information available on the Web is often inadequate and conducive to biased ideas and unrealistic expectations among patients. Patients often confront physicians with information on new clinical and surgical treatment. Unfortunately, most doctors are not prepared to discuss the matter with their patients, thus allowing suspicion and distrust to settle in to harm the physician-patient relationship^4^, ^5^, ^6^.

The websites most frequently looked up by parents/guardians were also the subject of interesting findings. Only a handful of them claimed they looked up specific ENT websites such as the one from ABORL-CCF.

We believe that at least partly the specialized websites lack content for the non-medical population and fail to offer information using clearer and more accessible language.

Most respondents used search engines such as GOOGLE and YAHOO when looking up medical information, as also found in American studies^10^, ^11^, ^12^. The most frequently used search keywords were:

a) ‘tonsillectomy’
b) ‘tonsil surgery’
c) ‘adenoid surgery’.

**Figure 1:**
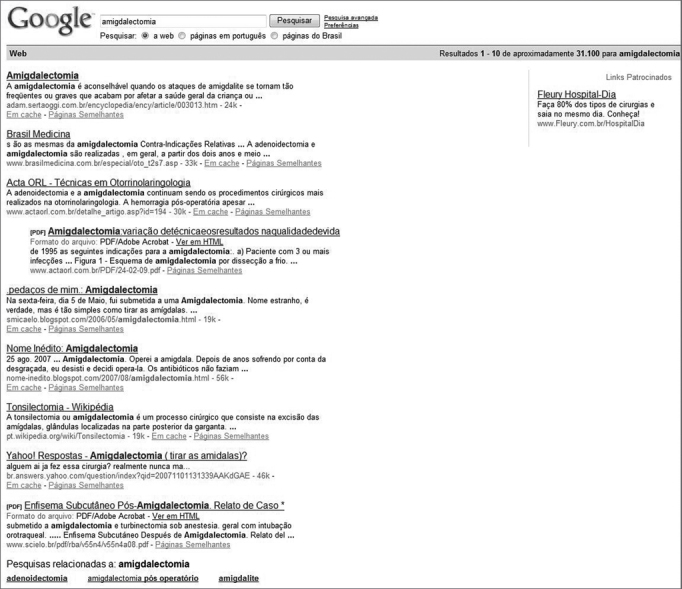
Top search results on GOOGLE for keyword 'tonsillectomy' (amigdelectomia).

**Figure 2:**
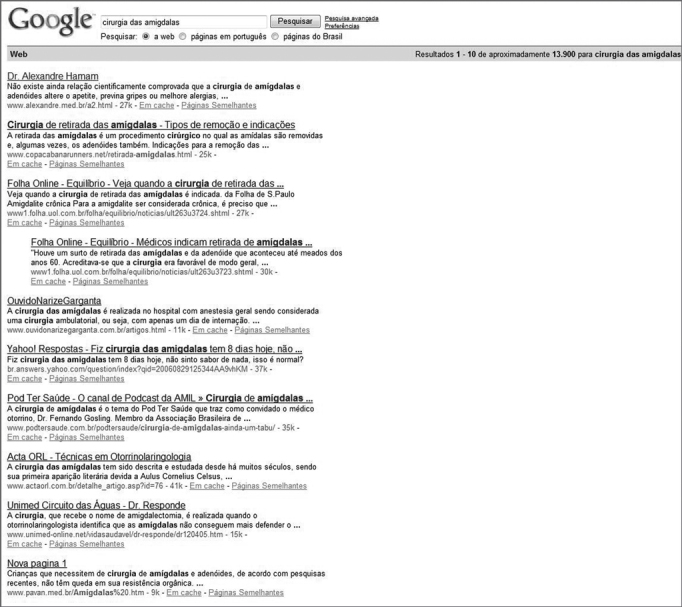
Top search results on GOOGLE for keyword 'tonsil surgery' (cirurgia das amígdalas).

**Figure 3:**
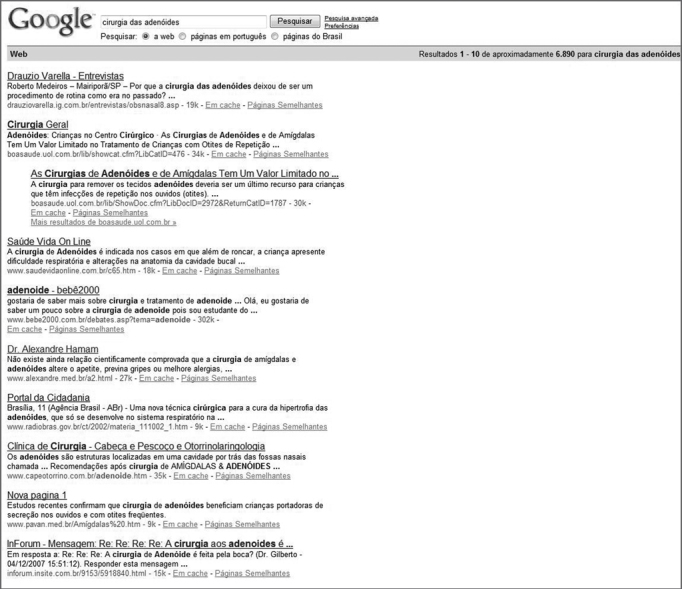
Top search results on GOOGLE for keyword 'adenoid surgery' (cirurgia das adenoides).

In April of 2008, GOOGLE showed 31,000 search results for 'tonsillectomy'; over 13,000 search results for 'tonsil surgery'; and over 6,000 search results for 'adenoid surgery'. Many of the search results did not derive from specific medical websites. We found patient websites, journals, and commercially available product web pages, among others. Figure 1, Figure 2, Figure 3 shows the search results for each of the keywords used on GOOGLE.

There is no specific legislation in Brazil over the publication of medical information on the Internet. Therefore, in 2001 the São Paulo State Medical (CREMESP) enacted an ordinance and published the 'Manual of Ethical Principles for Medical and Health Care Websites' to set the ground for the publication of medical information on the Web^13^.

The 'Manual of Ethical Principles for Medical and Health Care Websites' defines that the quality of a given website must be assessed according to the following parameters: transparency, honesty, quality of the information, free clarified consent, privacy, medical ethics, accountability, and origin^13^.

We assessed the 20 first search results on GOOGLE for each of the keywords according to the 'Manual of Ethical Principles for Medical and Health Care Websites' using parameters transparency (in relation to the purpose and identification of the person or entity responsible for the website), honesty (the goals of the website and the quality of the information provided in it in terms of accuracy, up-to-dateness, proper language, scientific backing, authors, and references).

None of the websites listed on these search results complied with the requirements set forth in the 'Manual of Ethical Principles for Medical and Health Care Websites'.

Commonly used engine searches such as GOOGLE and YAHOO list thousands of search results based on the keywords typed in by parents/guardians. These search results are picked by proprietary algorithms that collect data from thousands of websites on a daily basis and cross-reference the keywords against web pages found in the World Wide Web, to then yield a response containing the web pages with more similarities when compared to the chosen keywords.

All respondents allowed their children to be operated on. It is possible that the information gathered on the Web helped them make this decision, but the questionnaire was not answered by parents not opting for surgery for their children against medical recommendation.

Only 10% of respondents discussed the medical information they gathered on the Web with their ENT doctors, and only 2% of them were encouraged by their physicians to look up specific recommended websites on the Internet.

We believe that physicians must play a more proactive role in informing their patients of specific websites that contain accurate medical information. They should also discuss the information collected by their patients, answer their questions, and provide them with additional information when needed. Web pages featuring specific content and easy-to-read texts produced by specialists should be designed by ABORL-CCF to allow Brazilian ENT doctors to offer quality information to their patients.

Accurate parent and guardian preoperative guidance requires good understanding of the procedure to be carried out. Other forms of treatment, when available, alongside risk factors, benefits, and postoperative care must also be addressed. Alternative sources of information to parents and guardians are desired as a means to assist physicians in better informing their patients.

The Internet may be seen as a useful resource as it allows for the communication of information in interactive, multimedia forms. However, as the information available on the Web is not regulated, ENT doctors must become familiarized with reliable websites in order to recommend them to their patients.

Additional studies are required to better determine whether the information collected on the Web may be used to reduce preoperative anxiety, thus improving the postoperative satisfaction of both patients and their families.

## CONCLUSION

One hundred and seventeen (90%) of the 130 respondents with Internet access used the Web to search for information on their children's condition and treatment. Only 12 respondents (10%) discussed the information they gathered with the physician assigned to perform surgery. However, 91 (78%) reported that the information they collected impacted the decision made upon the procedure to be performed on their children. Most of the information was searched in non-medical generic websites such as GOOGLE and YAHOO, among others.

ENT physicians must be aware of the growing use of the Internet as a source of medical information and knowledgeable on the content of specific, easy to access, reliable websites to refer their patients to. Websites containing specific content, featuring opinions and documents written in a manner to make it easy for the reader to understand should be developed by ABORL-CCF to assist physicians in finding sources of information for their patients.
